# Tracking the polio virus down the Congo River: a case study on the use of Google Earth™ in public health planning and mapping

**DOI:** 10.1186/1476-072X-8-4

**Published:** 2009-01-22

**Authors:** Raoul Kamadjeu

**Affiliations:** 1 National Center for Immunization and Respiratory Diseases (NCIRD), Global Immunization Division (GID), Centers for Disease Control and Prevention (CDC), Atlanta, Georgia, 30333, USA

## Abstract

**Background:**

The use of GIS in public health is growing, a consequence of a rapidly evolving technology and increasing accessibility to a wider audience. Google Earth™ (GE) is becoming an important mapping infrastructure for public health. However, generating traditional public health maps for GE is still beyond the reach of most public health professionals. In this paper, we explain, through the example of polio eradication activities in the Democratic Republic of Congo, how we used GE Earth as a planning tool and we share the methods used to generate public health maps.

**Results:**

The use of GE improved field operations and resulted in better dispatch of vaccination teams and allocation of resources. It also allowed the creation of maps of high quality for advocacy, training and to help understand the spatiotemporal relationship between all the entities involved in the polio outbreak and response.

**Conclusion:**

GE has the potential of making mapping available to a new set of public health users in developing countries. High quality and free satellite imagery, rich features including Keyhole Markup Language or image overlay provide a flexible but yet powerful platform that set it apart from traditional GIS tools and this power is still to be fully harnessed by public health professionals.

## Background

*"Finishing the job of polio eradication is our best buy. We must do it. We are leaving a perpetual gift to generations of children to come" *WHO Director-General Dr. Margaret Chan [1].

Mapping in public health is not new. The cholera map by John Snow marked a critical turn in the use of maps to understand geographic patterns and disease [2]. Geographic Information System (GIS) technology is increasingly used by public health professionals, policy makers and other public health actors to better understand how geographic relationships affect disease transmission patterns [3-6], access to health care [7-11] and health outcome[12,13].

The use of GIS in public health is growing, a consequence of a rapidly evolving technology and increased accessibility to a wider audience. The recent development of web based mapping application like Google Maps™ (GE) [14] which provides free and easy access to geographic data and tools have contributed to the popularity of these new mapping approaches both within the general audience and among public health professionals.

The use of satellite images in public health has increased over time and covers areas as diverse as research, disaster management, outbreak response, emergency preparedness and humanitarian crisis [3,15-19]. Unfortunately, public health professionals still have difficulties in accessing relevant satellite data based on quality, time and cost [20]. GE is a virtual globe[21] that maps the earth by the superimposition of images obtained from satellites imagery and aerial photography. The availability of free satellites images in GE [22] has contributed to its popularity in the general public and among public health professionals. If GE is becoming a key mapping infrastructure for public health [23-29]; there are however, some characteristics that set it apart from traditional GIS software, notably its limited spatial data processing, analysis and modelling capabilities [27].

GE is packaged under two different licenses: Google Earth™, a free version with limited functionality and Google Earth Pro™ which has more functionality and is intended for commercial used. A third license called Google Earth Plus™ was discontinued [30]. All GE versions are available for use on personal computers running Microsoft Windows Operating systems, Mac OS X10.9 and above, Linux and FreeBSD [31].

### Google Earth™ imagery

Images in GE come from satellites and aerial photography (images from airplanes, kites and balloons). Some images are also provided by city or state governments. These images are not real time; the average age of high resolution images varies between 6 months to 5 years [32]. For this reason, GE images should be checked for accuracy when used for planning, because they may no reflect recent changes in landscape (new urban development, recent disasters etc.) Most lands are covered with a resolution of 15 meters per pixel and some populated centres of North America and Europe are covered with an even higher resolution (few meters by pixel), while oceans are covered with a much lower resolution, in the order of 500 m. In general the level of details of images in GE varies greatly; they tend to be more accurate in North America and Europe but are far less accurate when it comes to rural areas in developing countries. Clouds, shadows and heavy vegetal coverage also affect the visibility of some places of interest [31,32].

Because GE is primary a map viewer, it provides little functionality for creating traditional public health maps from scratch, although GE Pro™ allows the importation of the popular ESRI shape file (*.shp) and geospatial data (latitude and longitude) in tabular format [33]. Generating traditional public health maps with GE is still beyond the reach of most public health professionals, since this process requires a new set of skills (KML, geocoding) normally not prevalent in this group of professionals.

In this article, we describe how we used GE in public health planning, through the example of poliomyelitis (polio) eradication activities in the Democratic Republic of Congo (DRC) and we share the methods we used to generate public health maps for advocacy, training and to help understand the relationship between the entities involved in the polio outbreak and response. The method describes in this paper is applicable to all the current versions of GE and complement methods previously described to demonstrate the potential of these technologies and to help public health professionals and neogeographers face the "geo-rich society" [34,35].

### Polio eradication initiative and recent polio outbreaks in DRC

In 1988, the World Health Assembly voted to set the global goal for polio eradication [36]. Since then, the Global Polio Eradication Initiative remains the largest, internationally coordinated public health project. By the end of 2007, only 4 countries remained endemic for polio: Afghanistan, India, Pakistan and Nigeria [37] with persistent risks of exportation of the virus to polio free countries. This was the case in the Democratic Republic of the Congo (DRC), a country free from polio virus circulation since 2004, where 54 cases of polio caused by wild polio virus type 1 (WPV1) were reported in 4 provinces from February 2006 to December 2007 [37]. In response to this outbreak, mass immunization campaigns, also known as Supplemental Immunization Activities (SIA), were launched to interrupt the circulation of the virus and eliminate the risk of exportation; internally to unaffected provinces and externally to neighbouring countries. From the beginning of the outbreak in February 2006 to September 2007, 6 rounds of SIA, targeting all children aged 9 to 59 months and living in affected and high risk districts, were conducted. The initial impact of the campaign to alter the progression of the polio outbreak was low, as it seemed to progress north-east, following the course of the Congo River. A review of SIA activities conducted in August 2007 recommended specific strategies and actions to improve the quality of vaccination campaigns and consequently halt the circulation of WPV1. This resulted in the adoption of the "River Strategy", specifically targeting populations neighbouring the river. Since the beginning of 2008 the outbreak seems to be contained; by the time of this report, only one polio case due to WPV1 was reported, with onset of paralysis in February 2008.

### The Congo River and the polio outbreak

The Congo River is the second longest river in Africa (4700 km). Because it is readily navigable in sections, especially between Kinshasa and Kisangani, it is a major trade route and sees intense movement of persons and goods [38].

**Figure 1:**
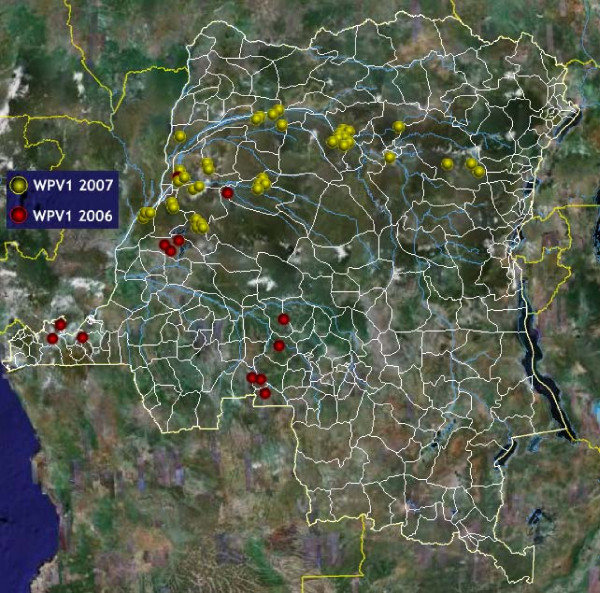
**Mapping wild polio virus cases in DRC**. The 40 cases of wild polio virus identified in DRC from January to December 2007 are depicted here. Cases seemed to follow the course of the Congo River. Since polio cases in the polio surveillance database are not geocoded, coordinates (Latitude and Longitude) of districts of residence of cases were extracted from the GeoNet name database and used as a proxy for case location on the map. Markers are custom Portable Network Graphics (PNG) images designed with Macromedia Fireworks™. Disclaimer: The map is intended solely to demonstrate the use of Google Earth™ in public health mapping. Any other intended use will be inappropriate. Refer to authoritative sources (World Health Organization) for polio eradication information.

The geospatial distribution of polio cases showed that the outbreak seemed to follow the course of the Congo River (Figure 1); this raised suspicion that the river could play an important role in the propagation of the outbreak to neighbouring districts. Detailed maps of entire sections of the river were not known or available, with the consequence that potential populations eligible for vaccination services and living on islands or along tributaries to the river were not included in immunization micro-plans, and therefore, not reached by routine vaccination or SIA services. The overall objective of the River Strategy was to stop the progression of WPV1 along the river by insuring that the entire eligible population living "on the river" was vaccinated against polio during the SIA. The groups targeted by the strategy included mobile populations on boats, canoes and rafts, populations of seasonal villages and fixed population on islands and stilt villages.

## Results

**Figure 2:**
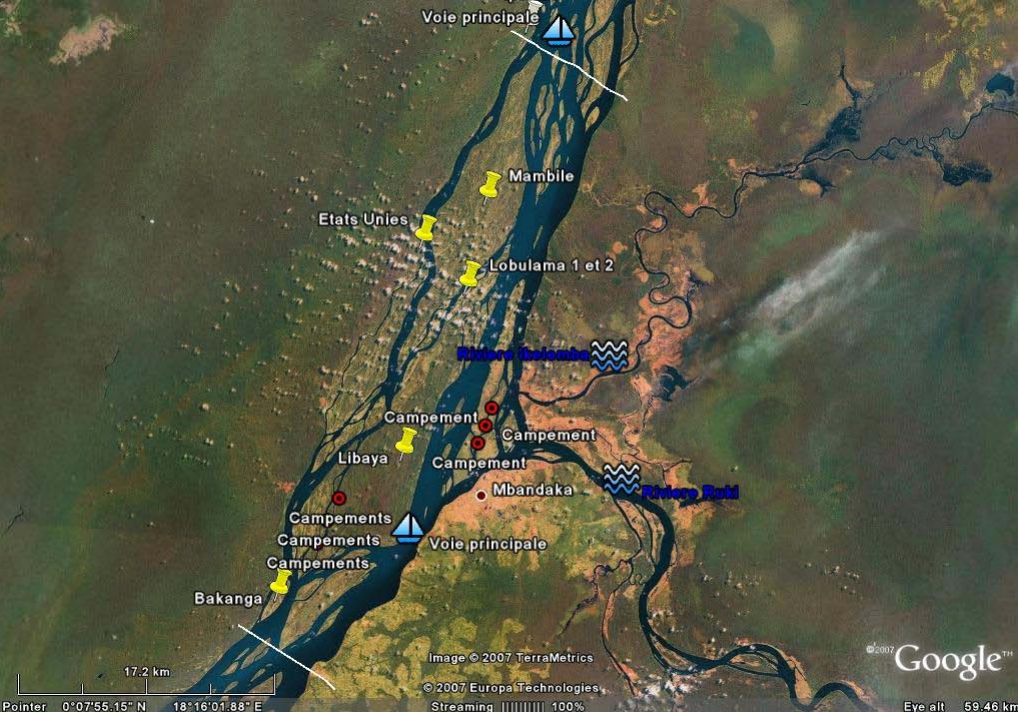
**Google Earth for public health mapping**. Google Earth allowed us to get maps of acceptable resolution to help elaborate the micro plan and guide operations on the field. This Google Map image of a portion of the Congo River around Mbandaka shows the main islands (yellow pins), known temporary (seasonal) settlements (read dots) and main navigation routes. The upper and lower white lines show the limits of district areas of responsibility.

The overall result of using GE was a better allocation of resources (fuel, outboard engines, and canoes) and an improved dispatch of vaccination teams (Figure 2). Social mobilization activities as well as supervision and monitoring were also greatly improved. With these improved micro-plans, populations previously missed by routine immunization services were identified and vaccinated. Vaccination teams were posted on passage points to screen all embarkations for eligible children; ports and shore-markets were continuously surveyed 24 h a day for arriving and departing ships during the entire duration of the campaign.

**Figure 3:**
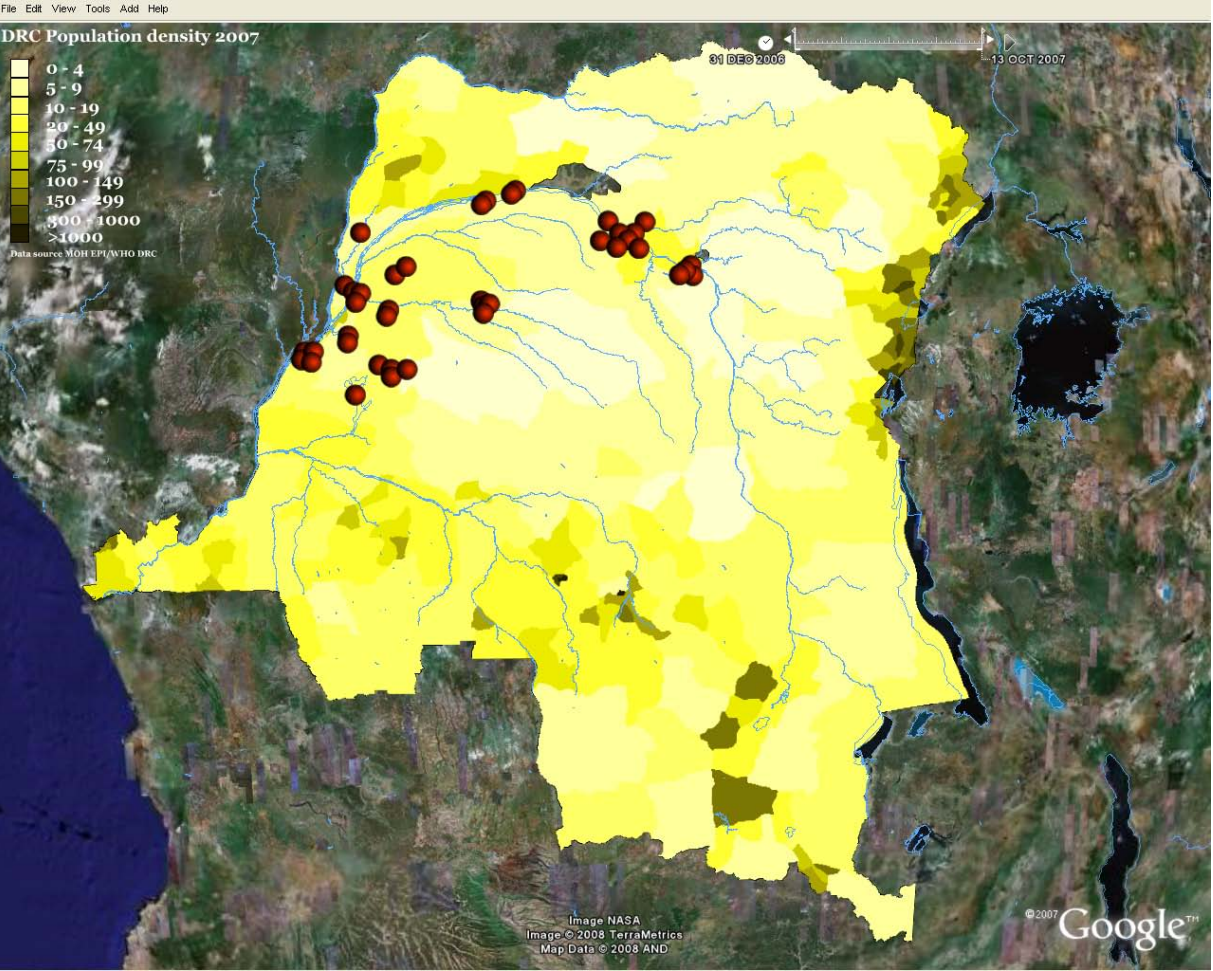
**Choropleth map of Population density, Congo River and Wild Polio virus cases**. KML layers resulted in high quality visual display and can improve our understanding of the spatiotemporal relationships between the entities involved in the polio outbreak. Colours and values for population density classes were chosen by the author and may not conform to existing standards. Markers are custom Portable Network Graphics (PNG) images designed with Macromedia Fireworks™. Disclaimer: The map is intended solely to demonstrate the use of Google Earth™ in public health mapping and has no other intentions whatsoever. Refer to authoritative sources for disease and population information about the Democratic Republic of Congo.

**Figure 4:**
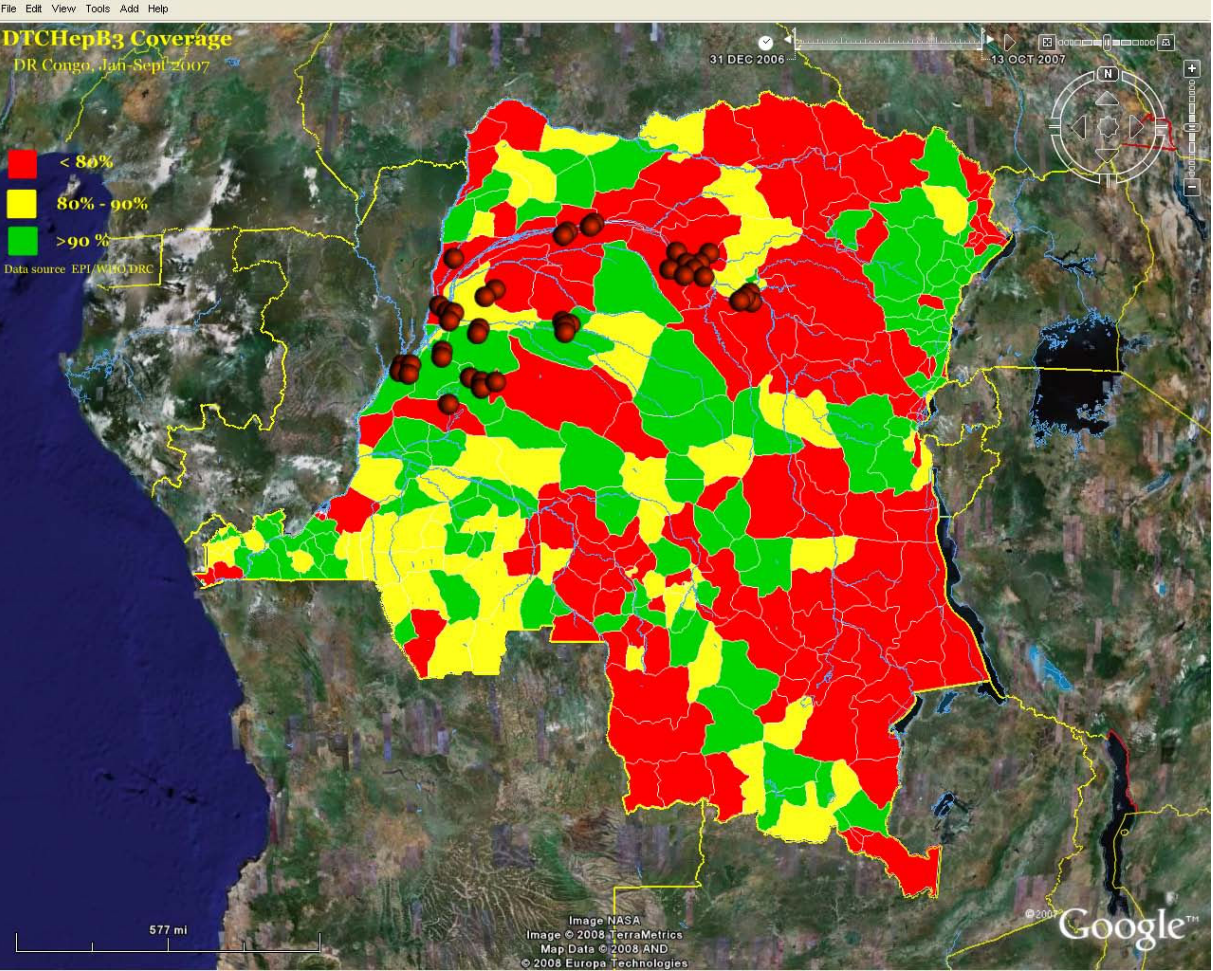
**Choropleth map of immunization coverage and Wild Polio virus cases**. KML layers resulted in high quality visual display and can improve our understanding of the spatiotemporal relationships between the entities involved in the polio outbreak. Colours and values for vaccination coverage classes were chosen by the author and may not conform to existing standards. Markers are custom Portable Network Graphics (PNG) images designed with Macromedia Fireworks™. Disclaimer: The map is intended solely to demonstrate the use of Google Earth™ in public health mapping and has no other intentions. Refer to authoritative sources for disease and immunization information about the Democratic Republic of Congo.

Figure 3 and 4 show traditional public health maps generated using GE. The primary purpose of using GE was to get an accurate view of the Congo River in order to improve the micro-planning process. The addition KML layers on existing GE images resulted in high quality visual display and improved our understanding of the spatiotemporal relationship between the entities involved in the polio outbreak. However, the technical skills required to turn GE into a powerful mapping tool is still beyond reach of the average public health professional. Meanwhile, district medical officers, surveillance officers and consultants were enthusiastic about the potential of GE; some of the users were able to direct the elaboration of an inventory of islands in their districts for future use. We expect the use of Google Earth in DRC to expand very rapidly beyond the area of immunization.

## Conclusion

GE images are not real time; this should be taken into consideration when deciding to use it as a source of image. It will be inappropriate to use it for features likely to quickly change over time (e.g. seasonal flooding, rapid urban development), because available images may not reflect the current reality. However GE can significantly improve our knowledge of the topography and provide key information for planning. Addition of KML layers and the use of other GE features provide an opportunity to design powerful visual effects for planning, teaching and advocacy.

GE has the potential of making mapping accessible to a new set of public health users in developing countries. High quality satellite imagery, rich features, including KML or image overlay provide a flexible but yet powerful platform that set it apart from traditional mapping tools.

## Methods

**Ethical consideration**s: the project did not directly involve human subjects; therefore, approval from an institutional review board was not sought.

Micro-planning is critical for successful implementation of polio SIA. It identifies population groups likely to be missed and recommends special activities to reach them with vaccination services. For the River Strategy, the micro planning process included key field players: district medical officers, consultants, community members, fishermen, boat drivers, and sailors.

### Google Earth™ and the Congo River

**Figure 5:**
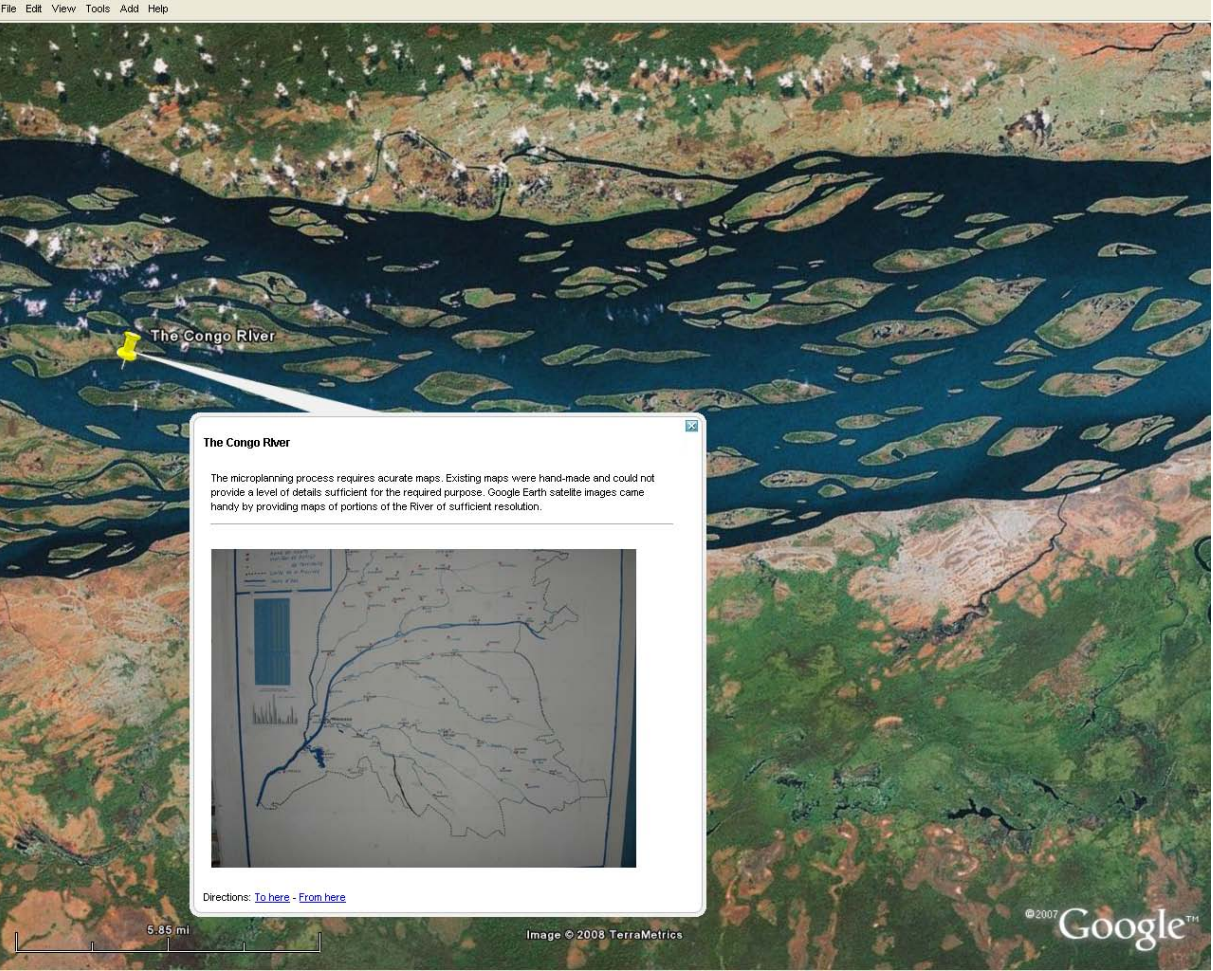
**Existing maps and Google Earth**. Existing maps were hand-made and could not provide a level of details sufficient for the required purpose. Google Earth satellite images came handy by providing maps of portions of the River of sufficient resolution to be used for planning purposes.

Successful implementation of any micro-plan requires widespread use of mapping. The biggest challenge was to find maps of portions of the river with acceptable resolution to help elaborate the micro-plan. In some parts of the world and mostly in developing countries, finding accurate maps can be difficult (Figure 5). The resolution of satellite imagery of rural areas of Africa provided by GE is generally very low and image quality can be further altered by clouds or heavy vegetal coverage of the rainy forest. Our objective however was to get overview of sections of the river, identify navigation passages and main islands in order to refine the micro planning process. GE, with its free global coverage of satellite imagery seemed like an interesting option to explore. By zooming on sections of the Congo River with GE, we were able to obtain images of the river's corridor of acceptable resolution. This improved view of the river, combined with the knowledge of local key informants, provided valuable information to better estimate distances, locate islands, and overall improve our knowledge of the topography. Administrative boundary layers were added to the maps using KML.

### KML implementation

The use of maps is well established in immunization. Hand-drawn maps have been used by vaccination teams on the ground to plan their daily activities; choropleth (colour coded) or composite maps have been used intensively to display vaccine preventable diseases and immunization coverage information across geographical units. GE has been used increasingly as a source of maps but the system is primary a viewer and provided limited options for creating traditional public health maps. Also, the skills require to dynamically generate complex KML map files using computer programming languages is still beyond the reach of public health professionals.

Keyhole Markup Language (KML) is an XML-based language for expressing geographic annotation and visualization on Web-based, two-dimensional maps and three-dimensional Earth browsers like Google Earth [39-41]. To generate thematic KML maps, we went through three basic steps:

### Step 1 – Conversion of shape file into KML

The ESRI shapefile (*.shp) stores geospatial vector data in a tabular format for GIS software. It is developed and regulated by ESRI [42] as an open specification for data interoperability among ESRI and other software products. A shape file is a set of three files that are mandatory to store the content of the shape file. The three mandatory files that make up the shapefile are: 1) the shape file (*.shp) format describing the feature geometry, 2) the shape index (*.shx) format storing the positional index of the geographic feature and 3) the data format (*.dbf) storing the columnar attribute for each shape in dBase III format [43].

We generated district and province boundary KML files by converting existing shape files into KML using a shape-to-kml converter [44]. The tool we used is a free utility that inputs shape file and outputs a KML file formatted based on user's specifications. Several shape-to-kml applications; both free and license exist but were not fully evaluated.

**Figure 6:**
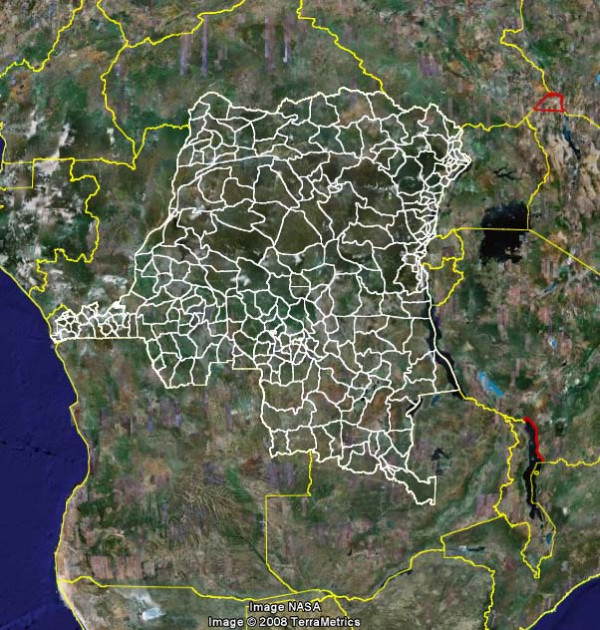
District boundaries for DRC resulting from the conversion of shape file (*.shp) into KML. Disclaimer: This map is intended solely to demonstrate the result of the conversion of shape into kml and should not be used for any other purposes. Refer to authoritative sources for country maps.

Figure 6 shows the output of the conversion of DRC shape file into KML, viewed with GE (additional file 1).

### Step 2 – Loading geographic feature names and coordinates in the database

**Figure 7:**
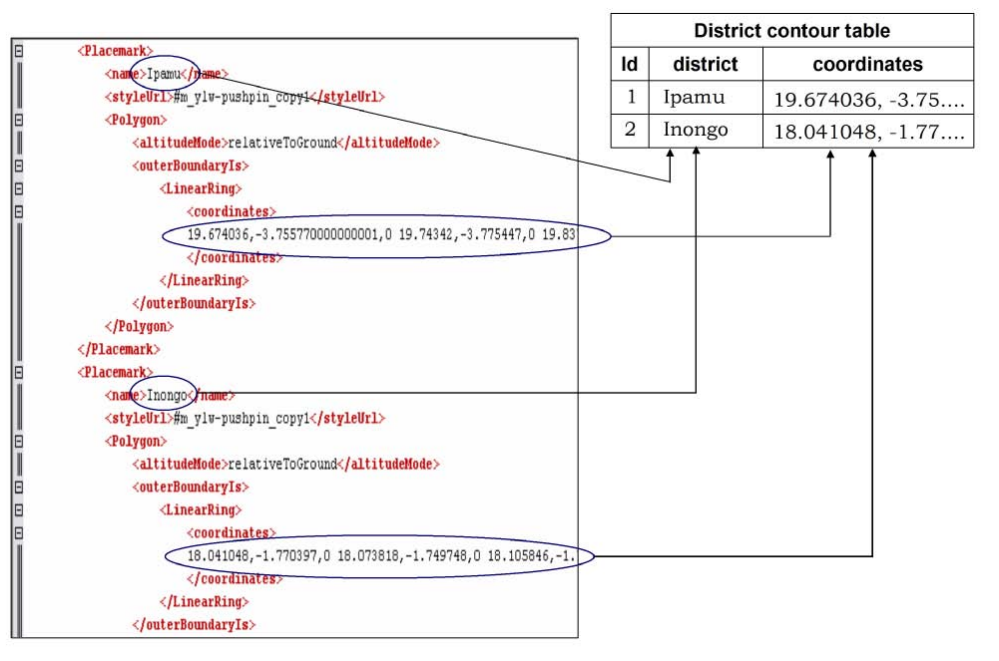
**Loading geographic information in the database**. A simple JavaScript routine parsed the KML Document Object Model (DOM), extracts values of **<name> **and **<coordinates> **tags and output a formatted text file which was secondarily stored in the database

A simple JavaScript routine (Additional file 2) parses the converted KML DOM (Document Object Model), extracts values of <name> and <coordinates> tags, and generates a formatted text file that can be stored in a database and used to dynamically generate custom KML (Figure 7)

### Step 3 – dynamic generation of KML using php

KML files were dynamically generated using simple scripts written with the web scripting language PHP [45]. Additional file 3 demonstrates how we used PHP to dynamically fill the content of KML polygons with colour based on the value of immunization coverage/population density for each district. In dot-mapping, polio cases were mapped using the lat/long coordinate of the district of residence of the case. To clustering avoid of points from the same districts (same latitude and longitude data), the first occurrence of the district's latitude and longitude was taken as the central coordinate around which subsequent identical points were randomly dispersed.

To explore and visualize the relationship of the current outbreak with impact measures e.g. natural structures, dot maps, choropleth maps and combinations of both were generated using available 2007 data (additional file 4).

### Data sources

Acute flaccid paralysis (AFP), routine immunization coverage and population data were obtained from the DRC Ministry of Health and the World Health Organization DRC country office. The data were reformatted and stored in the database hosted on a local Apache/PHP/MySQL server [45-48].

The DRC subset of the GEOnet Names Server (GSN)[49], a comprehensive database of foreign geographic feature names provided by the US National Geospatial Intelligence Agency [50] was used to extract coordinates (Latitudes and Longitudes) information for place names. Polio cases data are stored in the AFP database which includes location information (village, district and province of residence of case, but no geo-codes), clinical and laboratory information. Since the AFP database does not provide geo-codes, location of case was approximated to the most completely available geographical unit, which in this case was the district of residence of the case. Coordinates values for cases were obtained by linking the AFP database with the GSN database using the district of residence of cases

### Notes about GSN country file table

The GSN provides geographical names and geographic coordinates on DMS (Degrees – Minutes – Seconds) and decimal formats for a considerable number of geographic features and places around the world. The GSN table for DRC available for download on November 2008 consisted of 31834 records of geographic names information (tab separated text format), amenable to easy input into database management systems or spreadsheets. An overview of the GSN table shows that there can be more than one entry information for the same feature/place and more than one feature/place can have the same name. This lack of normalization makes the exploitation of the database prone to ambiguity and inconsistency; this should be kept in mind and acted upon when using any GSN dataset.

**Figure 8:**
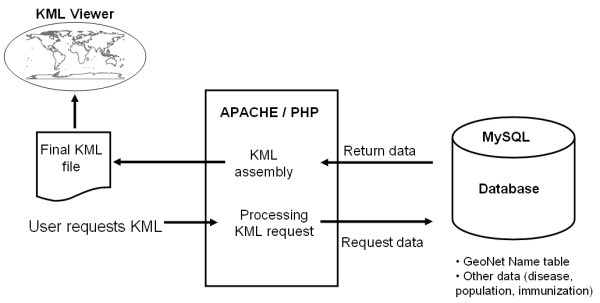
**System architecture**. A request for KML file is submitted by the client to the server. The MySQL database tier contains a subset table of the GeoNet Names, population and immunization data. Processing of the request involves querying the database to extract latitude and longitude data (case based mapping) or other required information (choropleth maps) and assembling the final KML. The final formatted KML file is returned to the client and is loaded in the viewer.

Figure 8 describes the architecture of the system. A request for KML file is submitted by the client to the server. The MySQL database tier contains a subset table of the GeoNet Names, population and immunization data. Processing of the request involves extraction querying the database to extract latitude and longitude data (case based mapping) or other required information (choropleth maps) and assembling the final KML. The final formatted KML file is returned to the client and is loaded into GE for viewing display in the viewer.

## Competing interests

The author declares that they have no competing interests.

## Authors' contributions

RK: performed data management and analysis, programmed the PHP routines used to generate KML and designed the visual content for the entire project.

## Supplementary Material

Additional file 1 **DRC kml district boundaries. **The file shows a sample output of shape-to-kml conversion using a shape-to-kml converter. (Open with Google Earth). Click here for file

Additional file 2 **JavaScript XML parser code. **This file shows the code of the JavaScript XML parser we used to extract values of kml tags <name> and <coordinates> to output a | separate file which can de secondarily loaded into a database (Open with a PDF reader). Click here for file

Additional file 3 **Density map php code generator**. This file shows the PHP code we used to generate population density kml map for DRC. (Open with a PDF reader). Click here for file

Additional file 4 **DRC population density map**. The file shows the population density map of the Democratic Republic of Congo dynamically generated with php. (Open with Google Earth). Click here for file
