# Supplementary material for: Tracking the polio virus down the Congo River: a case study on the use of Google Earth™ in public health planning and mapping
Source: Int J Health Geogr. 2009 Jan 22;8:4. doi: 10.1186/1476-072X-8-4 (PMC2645371; doi:10.1186/1476-072X-8-4)
Supplement: Additional file 2 — JavaScript XML parser code. This file shows the code of the JavaScript XML parser we used to extract values of kml tags and to output a | separate file which can de secondarily loaded into a database (Open with a PDF reader). [file 1476-072X-8-4-S2.pdf]

```
/*
By Dr Raoul Kamadjeu, Novermber 2008
Submitted as additional material for the IJHG article
This approach is quick and dirty and is just one of the mainy possible. So please
feel fre to use your own and even better method.
The bottom line is that you should be able to parse the xml, identify the tags of
interest and get thir values
------------------------------------------------------------------------------------
----------------------------
WHAT THIS SCRIPT
-----------------
The script parses the XML file (a kml file saved with the xml extension in this
case), extract the values of the <name> and <coordinate> tags and output a |
separated file that can be easily loaded in the database.

HOW TO USE THE SCRIPT
----------------------
Save you kml file as an xml file (*.xml).
Create an html file on your server (example: LoadKml.html). Make sure the xml is
in the same directory as the html

This first function reads the xml file in the memory.
=======FUNCTIOJN
LoadXMLDocument=====================================================
Copy this function on  js file (loadxmldoc.js)
link the html file to the javascript by inserting the <script
type="text/javascript" src="loadxmldoc.js"></script> in the head of the document.
*/
function loadXMLDocument(dname)
{
        var xmlDoc;
        // code for IE
        if (window.ActiveXObject)
        {
        xmlDoc=new ActiveXObject("Microsoft.XMLDOM");
        }
        // code for Mozilla, Firefox, Opera, etc.
        else if (document.implementation && document.implementation.createDocument)
        {
        xmlDoc=document.implementation.createDocument("","",null);
        }
        else
        {
        alert('Your browser cannot handle this script');
        }
        xmlDoc.async=false;
        xmlDoc.load(dname);
        return(xmlDoc);
}
/*
=======Script 2 =====================================================
Your final html file shoudl look like this
<html>
<head>
    <script type="text/javascript" src="loadxmldoc.js"></script>
</head>
```

```
<body>
<script type="text/javascript">
*/
    xmlDoc=loadXMLDocument("DRC.xml");
    //Indentifying the <name> tag inside the xml document
    var x=xmlDoc.getElementsByTagName('name');
    //indentifying the <coordinates> tag inside the xml document
    var y=xmlDoc.getElementsByTagName('coordinates');
    //we loop into all the <name> tags and for each of them, we pick the value for
<name> and <coordinates>
    for (i=0;i<x.length+1;i++)
    {
        document.write("<strong><font color='FF0000'>"); //the names will be
bolded to make them more visible
        document.write(x[i].childNodes[0].nodeValue);
        document.write("</font></strong>");
        document.write(" | "); // we use this separator to separate name and
coodinates for each of the placemark. This is just to improve readability.
        document.write(y[i].childNodes[0].nodeValue);
        document.write("<br />"); //Write each output on a separate line.
    }
/*</script>
</body>
</html>
The resulting file is a | seperated file that can be loaded into the database
```
