# Supplementary material for: Tracking the polio virus down the Congo River: a case study on the use of Google Earth™ in public health planning and mapping
Source: Int J Health Geogr. 2009 Jan 22;8:4. doi: 10.1186/1476-072X-8-4 (PMC2645371; doi:10.1186/1476-072X-8-4)
Supplement: Additional file 3 — Density map php code generator. This file shows the PHP code we used to generate population density kml map for DRC. (Open with a PDF reader). [file 1476-072X-8-4-S3.pdf]

```php
<?php
/*
Coded by Dr Raoul Kamadjeu
Submitted as additional material to IJHG submission
This is only one way of doing this.
*/
//connect to database
$conn = mysql_connect('localhost', 'root','') or die(mysql_error());
mysql_select_db('DBname');
?>
<html>
<head>

</head>

<body>

<?php

$kml="";
$kml .="
<?xml version='1.0' encoding='UTF-8'?>
<kml xmlns='http://earth.google.com/kml/2.2'>
<Document>
    <name>DRC Population Density Map.kml</name>
    <open>1</open>
    <Style id='grad1'>
        <LineStyle>
            <color>00ffffff</color>
        </LineStyle>
        <PolyStyle>
            <color>ffccffff</color>
        </PolyStyle>
    </Style>
    <Style id='grad2'>
        <LineStyle>
            <color>00ffffff</color>
        </LineStyle>
        <PolyStyle>
            <color>ff99ffff</color>
        </PolyStyle>
    </Style>
    <Style id='grad3'>
        <LineStyle>
            <color>00ffffff</color>
        </LineStyle>
        <PolyStyle>
            <color>ff66ffff</color>
        </PolyStyle>
    </Style>
    <Style id='grad4'>
        <LineStyle>
            <color>00ffffff</color>
        </LineStyle>
        <PolyStyle>
            <color>ff33ffff</color>
```

```
        </PolyStyle>
    </Style>
    <Style id='grad5'>
        <LineStyle>
            <color>00ffffff</color>
        </LineStyle>
        <PolyStyle>
            <color>ff00ecec</color>
        </PolyStyle>
    </Style>
    <Style id='grad6'>
        <LineStyle>
            <color>00ffffff</color>
        </LineStyle>
        <PolyStyle>
            <color>ff00cccc</color>
        </PolyStyle>
    </Style>
    <Style id='grad7'>
        <LineStyle>
            <color>00ffffff</color>
        </LineStyle>
        <PolyStyle>
            <color>ff04a4ac</color>
        </PolyStyle>
    </Style>
    <Style id='grad8'>
        <LineStyle>
            <color>00ffffff</color>
        </LineStyle>
        <PolyStyle>
            <color>ff03767c</color>
        </PolyStyle>
    </Style>
    <Style id='grad9'>
        <LineStyle>
            <color>00ffffff</color>
        </LineStyle>
        <PolyStyle>
            <color>ff014649</color>
        </PolyStyle>
    </Style>
    <Style id='grad10'>
        <LineStyle>
            <color>00ffffff</color>
        </LineStyle>
        <PolyStyle>
            <color>ff001f20</color>
        </PolyStyle>
    </Style>";
$kml .="<Folder>";
$kml .="<name>DRC population Density Map - 2007</name>";
/*Query database to get values for districts, coodinates and population density
based on value of density, we assign a specific color to the polygon by using the
kml <styleUrl> attributes which are define in the kml <style>
*/
```

```
$geoname="select district.district, district.coordinate, area.pdensity from
district,area where district.district=area.district";

     if ($result = mysql_query($geoname)) {
             if (mysql_num_rows($result)) {
                  while (list($district, $coordinate, $density) =
mysql_fetch_array($result)) {

                    $kml .="
                    <Placemark>
                        <name>$district</name>
                        <visibility>1</visibility>";
                        if($density<5){
                        $kml .="<styleUrl>#grad1</styleUrl>";
                        }
                        if($density>=5 && $density<10 ){
                            $kml .="<styleUrl>#grad2</styleUrl>";
                        }
                        if($density>=10 && $density<20 ){
                            $kml .="<styleUrl>#grad3</styleUrl>";
                        }
                        if($density>=20 && $density<50 ){
                            $kml .="<styleUrl>#grad4</styleUrl>";
                        }
                        if($density>=50 && $density<75 ){
                            $kml .="<styleUrl>#grad5</styleUrl>";
                        }
                        if($density>=75 && $density<100 ){
                            $kml .="<styleUrl>#grad6</styleUrl>";
                        }
                        if($density>=100 && $density<150 ){
                            $kml .="<styleUrl>#grad7</styleUrl>";
                        }
                        if($density>=150 && $density<300 ){
                            $kml .="<styleUrl>#grad8</styleUrl>";
                        }
                        if($density>=300 && $density<1000 ){
                            $kml .="<styleUrl>#grad9</styleUrl>";
                        }
                        if($density>=1000 ){
                            $kml .="<styleUrl>#grad10</styleUrl>";
                        }
                    $kml .="
                    <Polygon>
                            <altitudeMode>Absolute</altitudeMode>
                            <outerBoundaryIs>
                                <LinearRing>
                                <coordinates>$coordinate
                                </coordinates>
                            </LinearRing>
                            </outerBoundaryIs>
                    </Polygon>
                    </Placemark>
                    ";

                }
```

```
            }
    }

$kml .="</Folder>
    </Document>
</kml>";


//output the kml file
print $kml;

?>
</body>
</html>
```
