# Supplementary figures and images for: Tracking the polio virus down the Congo River: a case study on the use of Google Earth™ in public health planning and mapping
Source: Int J Health Geogr. 2009 Jan 22;8:4. doi: 10.1186/1476-072X-8-4 (PMC2645371; doi:10.1186/1476-072X-8-4)

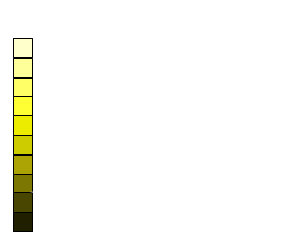

Supplement: Additional file 4 — DRC population density map. The file shows the population density map of the Democratic Republic of Congo dynamically generated with php. (Open with Google Earth). [file 1476-072X-8-4-S4.zip › files/gradient2.PNG]
